# Age-Related Variation in Odor Identification Performance in Children Assessed with the Brief Smell Identification Test

**DOI:** 10.3390/children13091239

**Published:** 2026-09-13

**Authors:** Taner Adıgüzel, Mehmet Ali Say

**Affiliations:** 1 Department of Pediatrics, Faculty of Medicine, Yalova University, 77200 Yalova, Türkiye; 2 Department of Otorhinolaryngology, Faculty of Medicine, Yalova University, 77200 Yalova, Türkiye; mehmet.say@yalova.edu.tr

**Keywords:** olfactory perception, smell, age factors, child, odor identification, brief smell identification test

## Abstract

**Background/Objectives:** Odor identification in children is influenced by age and developmental factors, yet age-related variation in Brief Smell Identification Test (B-SIT) performance remains insufficiently characterized. This study aimed to characterize age-related differences in B-SIT performance and examine its association with chronological age. **Methods:** This prospective cross-sectional study included 162 children aged 6–14 years recruited between January 2025 and January 2026. Participants completed the Turkish version of the B-SIT and were categorized as 6–8 years (*n* = 55), 9–11 years (*n* = 54), and 12–14 years (*n* = 53). B-SIT scores were compared using the Kruskal–Wallis test with Bonferroni-adjusted pairwise comparisons. Associations with age were assessed using Spearman correlation and simple linear regression. **Results:** Among the 162 children included in the study, B-SIT scores differed significantly among age groups (H(2) = 13.984, *p* = 0.001), with median scores of 9, 9, and 10, respectively. After Bonferroni correction, only the difference between the 6–8- and 12–14-year groups remained significant (*p* < 0.001). Age showed a modest positive association with B-SIT score (ρ = 0.325, *p* < 0.001). Regression analysis confirmed this association (B = 0.212, 95% CI: 0.117–0.308; β = 0.328; *p* < 0.001), with age explaining 10.8% of score variance (R^2^ = 0.108). **Conclusions:** B-SIT performance increases with age during childhood, although age explains only a limited proportion of individual variability. Age should therefore be considered when interpreting pediatric B-SIT scores.

## 1. Introduction

The olfactory system provides numerous functions that influence awareness of environmental hazards, eating behavior, and social communication [1]. Although olfactory function has been extensively characterized in adults, its assessment during childhood has received comparatively less attention due to shorter attention spans, developing linguistic abilities, lower odor experience, and a lack of age-appropriate standardized tests [2,3,4]. Unlike basic odor perception tasks, odor identification performance depends not only on olfactory perception but also on access to semantic memory, with performance shaped by prior experience and odor familiarity [5,6]. Accordingly, previous studies have consistently demonstrated that odor identification performance improves with increasing age during childhood [7,8].

The assessment of odor identification in children, however, presents methodological challenges that differ from those encountered in adults. Performance may be influenced by familiarity with the odorants and response alternatives, language and cognitive development, attention span, and the burden imposed by the testing procedure. Pediatric-specific instruments and adaptations of established adult tests have therefore been developed to minimize these influences. Studies using established psychophysical tests, including the University of Pennsylvania Smell Identification Test (UPSIT) and Sniffin’ Sticks, have shown age-dependent increases in odor identification scores in children, while pediatric instruments such as the NIH Toolbox Odor Identification Test have similarly shown poorer performance in younger children [7,9,10]. Among pediatric olfactory instruments, the Universal Sniff (U-Sniff) test was specifically developed for children and validated in a multicenter study involving 19 countries, including Türkiye [11]. Subsequent international studies further supported its reliability and expanded its normative database across additional populations [12]. Importantly, differences between testing instruments may themselves influence performance; for example, children have been reported to perform differently on UPSIT and Sniffin’ Sticks despite completing both tests successfully [9]. Thus, age-related findings obtained with one odor identification instrument cannot necessarily be assumed to apply directly to another.

The Brief Smell Identification Test (B-SIT), originally developed as the 12-item Cross-Cultural Smell Identification Test, is an abbreviated odor identification measure derived from the 40-item UPSIT [13]. Its standardized scratch-and-sniff format enables odor identification to be assessed within a brief and straightforward testing procedure. However, children’s olfactory recognition performance is strongly influenced by age, odor familiarity, and cognitive/linguistic development [14,15], which may complicate the interpretation of B-SIT scores. Although pediatric-specific instruments such as the U-Sniff are available, age-related variation in B-SIT performance during childhood remains insufficiently characterized. The present study was not designed to introduce or validate a new pediatric olfactory test; rather, it aimed to characterize age-related variation in performance on the existing B-SIT, a brief and standardized odor identification measure available in a Turkish-language version and previously used in Turkish clinical populations [16]. Characterizing age-related B-SIT performance may therefore provide instrument-specific information relevant to its interpretation in children.

Specifically, we compared B-SIT total scores across predefined age groups and examined the association between chronological age and B-SIT performance across the study population. We hypothesized that B-SIT performance would increase with age, with older children demonstrating higher odor identification scores than younger children.

## 2. Materials and Methods

### 2.1. Study Design and Participants

This prospective cross-sectional study protocol was approved by the Ethics Committee of Yalova University (approval no. 2024/257, date: 6 November 2024). The study was conducted in accordance with the principles of the Declaration of Helsinki. Written informed consent was obtained from the parents or legal guardians of all participants before enrollment. This single-center study included children aged 6–14 years recruited from the pediatric outpatient clinic of Yalova Training and Research Hospital between January 2025 and January 2026. The study population consisted of children attending the pediatric outpatient clinic who had no known olfactory dysfunction or known condition affecting olfactory function. All participants underwent an otorhinolaryngological examination before olfactory testing to identify conditions that could potentially interfere with odor perception or odor identification performance.

Children with known nasal or sinonasal pathology, an active or recent upper respiratory tract infection, congenital syndromes, a history of head trauma, neurodegenerative disease, or autoimmune disease were excluded from the study. During the pre-test otorhinolaryngological evaluation, children with clinically evident nasal or sinonasal conditions that could interfere with olfactory function were considered ineligible for olfactory assessment. Participants who were unable to understand or adequately complete the odor identification procedure were also excluded from the final analysis.

A total of 162 children who fulfilled the eligibility criteria and completed the olfactory assessment were included in the final analysis. Demographic characteristics, including age and sex, were recorded for each participant.

### 2.2. Olfactory Assessment

Odor identification performance was assessed using the Turkish-language version of the Brief Smell Identification Test (B-SIT^®^; Sensonics International, Haddon Heights, NJ, USA). The Turkish version of the B-SIT was selected because of its standardized, brief, and easily administered format, which was considered practical for use in a pediatric outpatient setting. The B-SIT consists of 12 microencapsulated odorants presented in a scratch-and-sniff format. Each odorant is accompanied by four response alternatives, only one of which represents the correct response.

All olfactory assessments were administered individually by the same investigator to minimize inter-examiner variability. For each item, the odorant was released according to the standardized scratch-and-sniff procedure and presented to the participant. Because the study population consisted of children, the four response alternatives were read aloud by the investigator in a standardized manner to minimize the potential influence of differences in reading ability. Participants were then asked to select the response that best represented the perceived odor.

Each correctly identified odor was assigned one point, yielding a total B-SIT score ranging from 0 to 12, with higher scores indicating better odor identification performance. The B-SIT total score was analyzed as a continuous measure of odor identification performance rather than being categorized according to adult diagnostic cutoff values.

### 2.3. Age Groups and Outcome Measures

The primary outcome of the study was the B-SIT total score. For age-stratified analyses, participants were categorized into three consecutive 3-year age bands: 6–8 years, 9–11 years, and 12–14 years. This grouping was used to characterize age-related differences across the younger, intermediate, and older portions of the study age range while maintaining comparable sample sizes across groups. In addition to this categorical approach, chronological age was analyzed as a continuous variable to avoid relying solely on age-group cutoffs and to evaluate the overall association between age and B-SIT performance without the loss of information associated with age categorization. Sex was recorded as a demographic variable, and its distribution was compared across the three age groups to assess group comparability.

### 2.4. Sample Size Considerations

A sensitivity power analysis was performed based on the final sample of 162 participants. For a three-group comparison, assuming an α level of 0.05 and 80% power, the available sample size was sufficient to detect a minimum effect size of approximately Cohen’s f = 0.25, corresponding to a moderate effect. Because the primary between-group analysis was performed using the non-parametric Kruskal–Wallis test, this sensitivity analysis was considered an approximate assessment of sample-size adequacy.

### 2.5. Statistical Analysis

Statistical analyses were performed using IBM SPSS Statistics for Windows, version 25.0 (IBM Corp., Armonk, NY, USA). Continuous variables were summarized as mean ± standard deviation (SD) and/or median with interquartile range (IQR), as appropriate, whereas categorical variables were presented as numbers and percentages. Age-specific descriptive statistics were additionally calculated for each individual year of age from 6 to 14 years and are presented in Supplementary Table S1. The distribution of continuous variables was assessed using the Shapiro–Wilk test. As B-SIT total scores were not normally distributed across the age groups, non-parametric methods were used for group comparisons. Differences in B-SIT total scores among the three age groups (6–8, 9–11, and 12–14 years) were evaluated using the Kruskal–Wallis test. The effect size for the Kruskal–Wallis test was estimated using epsilon-squared (ε^2^). When an overall significant difference was detected, pairwise comparisons were performed using the Mann–Whitney U test. To account for multiple comparisons, Bonferroni correction was applied, resulting in an adjusted significance threshold of α = 0.0167 (0.05/3) for pairwise comparisons. Differences in sex distribution among age groups were assessed using the Pearson chi-square test. Sex differences in B-SIT total scores were evaluated using the Mann–Whitney U test in the overall cohort and separately within each predefined age group. The association between age and B-SIT total score was evaluated using Spearman’s rank correlation coefficient. In addition, simple linear regression analysis was performed with B-SIT total score as the dependent variable and age as the independent variable to quantify the change in B-SIT score associated with increasing age. As a sensitivity analysis, multiple linear regression was performed with age and sex entered simultaneously as independent variables and B-SIT total score as the dependent variable to determine whether the association between age and B-SIT performance remained after adjustment for sex. Regression results were reported as unstandardized regression coefficients (B), standardized coefficients (β), 95% confidence intervals (CI), and coefficients of determination (R^2^). All statistical tests were two-tailed, and *p* < 0.05 was considered statistically significant, except for Bonferroni-adjusted pairwise comparisons.

## 3. Results

A total of 162 children aged 6–14 years were included in the study. The mean age of the participants was 9.96 ± 2.59 years, and 83 (51.2%) were female. Participants were distributed relatively evenly across the three predefined age groups: 55 (34.0%) were aged 6–8 years, 54 (33.3%) were aged 9–11 years, and 53 (32.7%) were aged 12–14 years. Sex distribution did not differ significantly among the age groups (*p* = 0.508) (Table 1).

The median B-SIT total score in the overall cohort was 9 (IQR, 3), with scores ranging from 5 to 12. B-SIT performance increased across age groups, with median scores of 9 (IQR, 2), 9 (IQR, 3), and 10 (IQR, 2) in the 6–8-, 9–11-, and 12–14-year groups, respectively. The overall difference in B-SIT scores among the three age groups was statistically significant (Kruskal–Wallis H(2) = 13.984, *p* = *0*.001, ε^2^ = 0.087) B-SIT total scores did not differ significantly between female and male participants in the overall cohort (*p* = 0.201) or within the 6–8-year (*p* = 0.736), 9–11-year (*p* = 0.424), and 12–14-year (*p* = 0.369) age groups (Table 1).

In pairwise analyses, children aged 6–8 years had lower B-SIT scores than those aged 9–11 years (U = 1143.5, Z = −2.106, *p* = 0.035) and 12–14 years (U = 854.5, Z = −3.774, *p* < 0.001), whereas the difference between the 9–11- and 12–14-year groups was not significant (U = 1193.5, Z = −1.506, *p* = 0.132). After applying the Bonferroni-adjusted significance threshold (α = 0.0167), only the difference between the 6–8- and 12–14-year groups remained statistically significant (Table 2).

**Figure 1:**
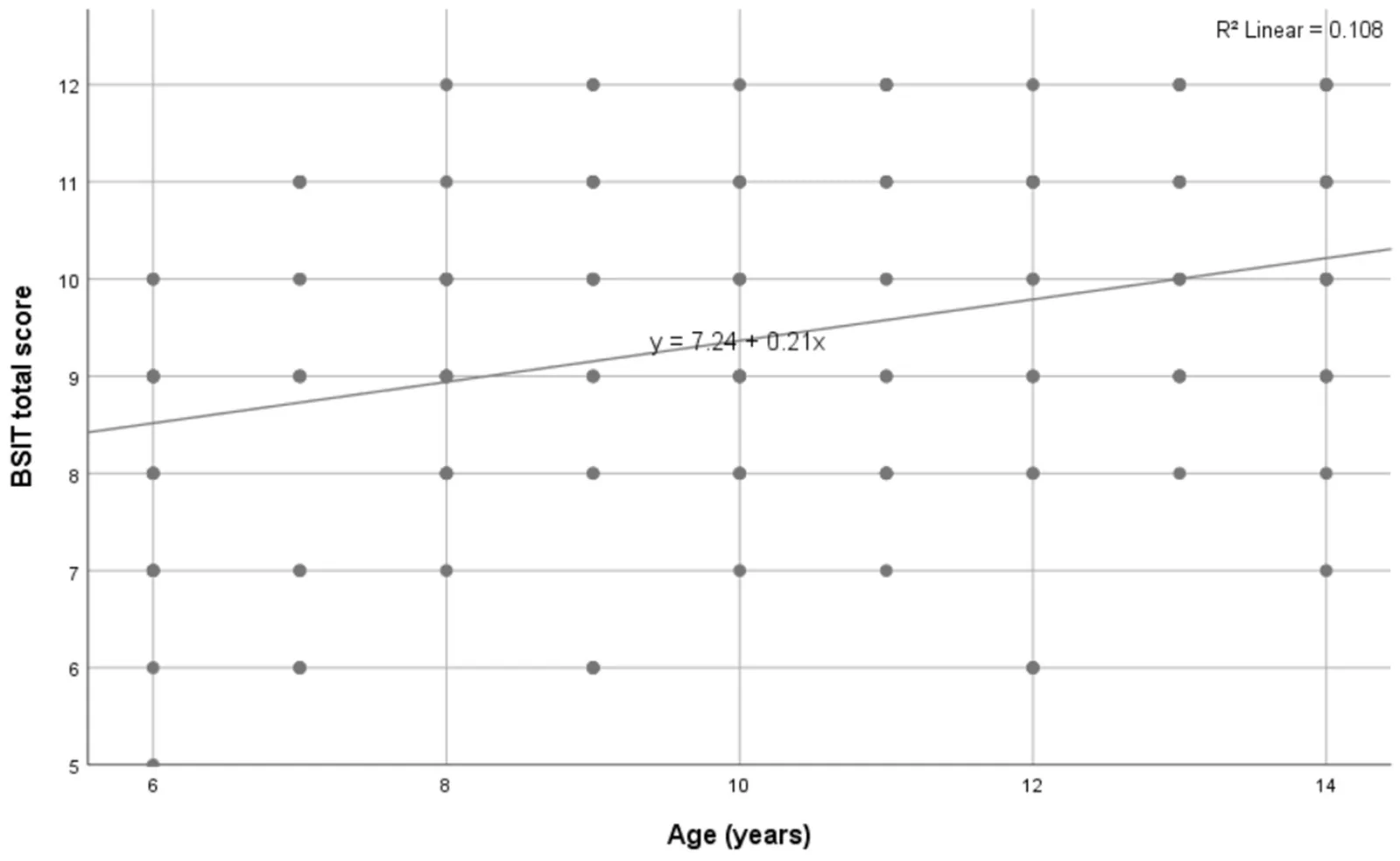
Association between age and Brief Smell Identification Test (B-SIT) total score in children (*n* = 162). The solid line represents the fitted linear regression line. The fitted regression equation was y = 7.24 + 0.21 × age. Age was positively associated with B-SIT total score (B = 0.212, 95% CI: 0.117–0.308, R^2^ = 0.108, *p* < 0.001).

Age was positively correlated with B-SIT total score (Spearman’s ρ = 0.325, *p* < 0.001). Consistent with this finding, simple linear regression demonstrated a significant positive association between age and B-SIT performance (B = 0.212, 95% CI, 0.117–0.308; β = 0.328; *p* < 0.001). The regression model was statistically significant (F(1,160) = 19.326, *p* < 0.001) and explained 10.8% of the variance in B-SIT total scores (R^2^ = 0.108) (Table 3 and Figure 1). In a sensitivity analysis including age and sex simultaneously, the overall multiple linear regression model was significant (F(2,159) = 10.897, *p* < 0.001, R^2^ = 0.121). Age remained independently associated with B-SIT total score after adjustment for sex (B = 0.204, 95% CI: 0.108–0.299, β = 0.315, *p* < 0.001), whereas sex was not significantly associated with B-SIT total score (B = −0.380, 95% CI: −0.874–0.114, β = −0.114, *p* = 0.131) (Table 3).

**Figure 2:**
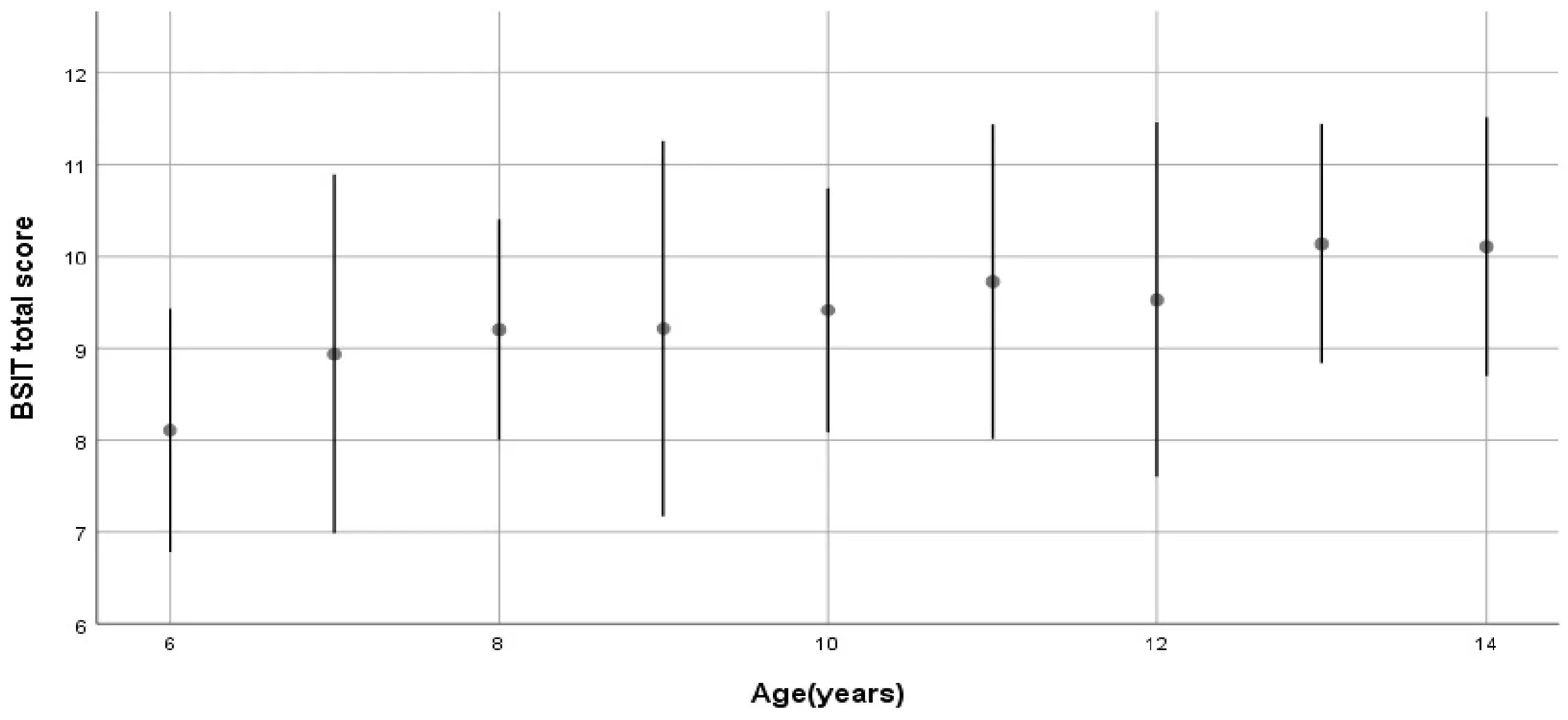
Age-specific mean Brief Smell Identification Test (B-SIT) scores in children. Error bars represent ±1 standard deviation. The number of participants at ages 6, 7, 8, 9, 10, 11, 12, 13, and 14 years was *n* = 19, 16, 20, 19, 17, 18, 19, 15, and 19, respectively.

Age-specific descriptive statistics for each individual year of age, including mean ± SD and median (IQR) B-SIT scores, are presented in Supplementary Table S1. Age-specific mean B-SIT scores showed an overall upward pattern across the 6–14-year age range, although the increase was not strictly monotonic at every individual age. Figure 2 illustrates the age-specific mean B-SIT scores, with error bars representing ±1 standard deviation.

Item-level correct response rates for the 12 B-SIT items are presented in Table 4. In the overall cohort, correct response rates ranged from 69.8% to 85.8%. Variation across age groups was also observed for individual items, most notably for Item 9, for which the correct response rate increased from 49.1% in children aged 6–8 years to 75.9% in those aged 9–11 years and 84.9% in those aged 12–14 years.

## 4. Discussion

The principal finding of the present study was that odor identification performance assessed with the B-SIT varied with age in children. Older children generally achieved higher B-SIT scores than younger children, and the most robust between-group difference was observed between the youngest and oldest age groups. When age was analyzed as a continuous variable, it was positively associated with B-SIT performance, further supporting an age-related pattern in odor identification. Although differences between all age groups did not reach statistical significance, the overall pattern indicated higher odor identification performance with increasing age. Taken together, these findings support a positive association between age and B-SIT performance during childhood. Importantly, these findings should not be interpreted as establishing pediatric-specific normative thresholds or diagnostic cutoff values for the B-SIT.

The observed age-related pattern is consistent with previous pediatric studies using other psychophysical odor identification tests [7,8]. Studies employing the UPSIT, Sniffin’ Sticks, and pediatric-specific odor identification instruments have generally demonstrated better identification performance with increasing age [9,17]. These observations support the concept that interpretation of odor identification scores in children requires consideration of age. However, performance on odor identification tests cannot be directly equated across different instruments, because tests differ in the number and type of odorants, response alternatives, administration procedures, and the degree of cultural familiarity with the presented odors [18,19]. The present study adds to the existing pediatric literature by providing age-specific data on odor identification performance using the B-SIT.

Several mechanisms may contribute to the association between age and odor identification performance. Odor identification is a higher-order cognitive task that extends beyond the perception of an odor stimulus, requiring the recognition of odor quality, semantic processing of odor-related associations, retrieval of lexical information from memory, and selection of the appropriate response among alternatives [5,20,21]. Age-related differences in children’s odor identification may reflect the gradual accumulation of odor-specific semantic knowledge, expanding vocabulary, and the strengthening of associations between olfactory percepts and verbal labels, rather than maturation of basic olfactory sensitivity [22,23,24,25]. The concurrent maturation of semantic knowledge, executive functioning, and working memory supports age-related differences in children’s forced-choice odor identification performance [5,23]. Accordingly, the higher B-SIT scores observed in older children may reflect not only differences in olfactory function but also age-related differences in cognitive, semantic, and odor-related experience involved in odor identification. In the present study, response alternatives were read aloud in a standardized manner by the same investigator, which may have reduced the potential influence of differences in reading ability on test performance, particularly among younger children. However, reading the response alternatives aloud does not eliminate the potential influence of linguistic and semantic factors, such as vocabulary, semantic knowledge, and familiarity with odor labels, on forced-choice odor identification performance.

An important observation was that the age-related increase in B-SIT performance was not strictly monotonic across individual ages, and significant differences were not observed between all age groups after correction for multiple comparisons. Although the overall difference among age groups was significant, only the comparison between children aged 6–8 years and those aged 12–14 years remained significant after Bonferroni correction. Similarly, although chronological age was significantly associated with B-SIT performance, it explained only 10.8% of the variance in total scores. These findings suggest that the relationship between age and odor identification performance during childhood is evident at the group level but does not translate into clear separation between successive age groups. The limited proportion of variance explained by age also indicates substantial individual variability in B-SIT performance among children of similar ages. Such variability may reflect differences in odor familiarity, cultural and environmental exposure, and cognitive or verbal abilities, although these factors were not directly assessed in the present study.

Objective assessment of olfactory function is also clinically relevant in rhinological disorders, as olfactory impairment has been associated with disease characteristics and inflammatory endotypes in chronic rhinosinusitis, supporting the importance of standardized olfactory assessment across different age groups and clinical settings [26]. In the pediatric context, the B-SIT has characteristics that may make it practical for pediatric olfactory assessment, including its brief administration time and forced-choice format. Nevertheless, the present findings indicate that age should be considered when interpreting B-SIT scores in children. For example, an apparently low B-SIT score in a younger child may partly reflect age-related variation in odor identification performance and should not automatically be interpreted as evidence of pathological olfactory dysfunction. Conversely, the same score in an older child may merit greater clinical attention when interpreted in the appropriate clinical context. Applying a single age-independent interpretation throughout childhood may obscure expected age-related variation, particularly when younger and older children are compared. At the same time, the considerable within-age variability observed in the present cohort argues against interpreting a child’s B-SIT score solely on the basis of chronological age. Accordingly, chronological age alone has limited value for predicting the expected B-SIT score of an individual child. The present findings should therefore be regarded as a characterization of age-related B-SIT performance rather than as definitive pediatric normative or diagnostic reference values. Establishing such reference values would require larger and more representative pediatric samples, ideally with sufficient numbers at each individual age and assessment of additional factors potentially affecting odor identification performance.

Several strengths of the present study should be acknowledged. The study included 162 children who were evenly distributed across the predefined age groups, allowing age-related differences to be examined without substantial imbalance in group sizes. Olfactory assessment was standardized and performed by the same investigator, with response alternatives read aloud to minimize the potential influence of reading ability. In addition, the Turkish version of the B-SIT was used throughout the study. Age was analyzed both categorically and continuously, providing complementary perspectives on its association with B-SIT performance. The use of correction for multiple comparisons further strengthened the interpretation of between-group differences.

The findings should also be interpreted in light of several limitations. First, the study had a cross-sectional design; therefore, the observed differences between age groups cannot be interpreted as direct evidence of longitudinal developmental change within individual children. Second, the study was conducted in a single-center pediatric outpatient population rather than a community-based pediatric population. This clinic-based recruitment may have introduced selection bias and may limit the generalizability of the findings to the broader pediatric population and to children from different geographic, cultural, or socioeconomic backgrounds. Although the Turkish version of the B-SIT was used, odor identification performance may still be influenced by individual differences in odor familiarity and cultural or environmental exposure. Furthermore, although pediatric-specific instruments such as the Sniffin’ Kids test are available, the Turkish version of the B-SIT has not, to our knowledge, been specifically validated in children aged 6–14 years. This should be considered when interpreting the present findings. In addition, test–retest reliability was not assessed in the present cohort because repeated B-SIT administration was not included in the original study protocol. Therefore, the reproducibility of B-SIT scores in this pediatric population remains to be evaluated in future studies. Third, cognitive, language, and odor-familiarity measures were not independently assessed, preventing determination of their relative contributions to B-SIT performance. The relatively modest variance explained by chronological age suggests that such unmeasured factors may be relevant. Finally, the present sample was not designed to establish population-based pediatric normative values or diagnostic cutoff scores. Future multicenter studies involving larger and more diverse pediatric populations, with adequate representation at each individual age and assessment of relevant cognitive, linguistic, and cultural factors, are warranted to establish age-specific reference data and determine the clinical utility of the B-SIT in pediatric olfactory assessment.

## 5. Conclusions

In conclusion, B-SIT performance was positively associated with age in children, with older children generally demonstrating higher odor identification scores than younger children. However, chronological age explained only a modest proportion of the variability in B-SIT performance, highlighting substantial individual variation among children of similar ages. These findings support consideration of age when interpreting pediatric B-SIT scores, while indicating that age alone does not fully account for differences in odor identification performance. The present findings characterize age-related patterns in pediatric B-SIT performance but should not be interpreted as normative or diagnostic reference values. Larger, multicenter studies with adequate representation across individual ages are needed to establish age-specific reference data and further define the clinical utility of the B-SIT in pediatric olfactory assessment.

## Figures and Tables

**Table 1:** Demographic and olfactory characteristics of the study population according to age groups.

Characteristic	Total (*n* = 162)	6–8 Years (*n* = 55)	9–11 Years (*n* = 54)	12–14 Years (*n* = 53)	*p* Value
**Age, years, mean ± SD**	9.96 ± 2.59	7.02 ± 0.85	9.98 ± 0.84	13.00 ± 0.85	—
**Sex, n (%)**					0.508 ^a^
Female	83 (51.2)	25 (45.5)	28 (51.9)	30 (56.6)	
Male	79 (48.8)	30 (54.5)	26 (48.1)	23 (43.4)	
**B-SIT total score, median (IQR)**	**9 (3)**	**9 (2)**	**9 (3)**	**10 (2)**	**0.001 ^b^**
Mean ± SD	9.36 ± 1.67	8.75 ± 1.54	9.44 ± 1.71	9.91 ± 1.58	
Range	5–12	5–12	6–12	6–12	
**B-SIT total score by sex, median (IQR)**					
Female	9 (2)	9 (2)	9 (2)	10 (3)	
Male	9 (3)	9 (3)	9.5 (3)	10 (2)	
**Female vs. male, *p* value ^c^**	0.201	0.736	0.424	0.369	

^a^ Pearson chi-square test. ^b^ Kruskal–Wallis test. ^c^ Mann–Whitney U test comparing B-SIT total scores between female and male participants. B-SIT, Brief Smell Identification Test; IQR, interquartile range; SD, standard deviation. Bold text indicates main characteristic headings; bold *p* values indicate statistically significant results (*p* < 0.05).

**Table 2:** Pairwise comparisons of B-SIT total scores among age groups.

Comparison	Mann–Whitney U	Z	*p* Value
6–8 vs. 9–11 years	1143.5	−2.106	0.035
6–8 vs. 12–14 years	854.5	−3.774	<0.001 *
9–11 vs. 12–14 years	1193.5	−1.506	0.132

Overall Kruskal–Wallis: H(2) = 13.984, *p* = 0.001. Pairwise comparisons were evaluated using a Bonferroni-corrected significance threshold of α = 0.0167. Only the 6–8 vs. 12–14 years comparison remained statistically significant after correction. * Statistically significant after Bonferroni correction (adjusted significance threshold, α = 0.0167).

**Table 3:** Association of age and sex with B-SIT total score.

Analysis	Spearman’s ρ	B	95% CI for B	β	R^2^	Test Statistic	*p* Value
Spearman correlation	0.325	—	—	—	—	—	**<0.001**
Linear regression: age	—	0.212	0.117–0.308	0.328	0.108	F(1,160) = 19.326	**<0.001**
Multiple linear regression model	—	—	—	—	0.121	F(2,159) = 10.897	**<0.001**
Age	—	0.204	0.108–0.299	0.315	—	—	**<0.001**
Sex	—	−0.380	−0.874–0.114	−0.114	—	—	0.131

B, unstandardized regression coefficient; β, standardized regression coefficient; CI, confidence interval; R^2^, coefficient of determination; B-SIT, Brief Smell Identification Test. The multiple linear regression model included age and sex simultaneously as independent variables. Sex was coded as 0 = female and 1 = male. Bold *p* values indicate statistically significant results (*p* < 0.05).

**Table 4:** Correct response rates for individual B-SIT items in the overall cohort and according to age group.

B-SIT Item	Overall (*n* = 162), *n* (%)	6–8 Years (*n* = 55), *n* (%)	9–11 Years (*n* = 54), *n* (%)	12–14 Years (*n* = 53), *n* (%)
**Item 1**	126 (77.8)	41 (74.5)	41 (75.9)	44 (83.0)
**Item 2**	121 (74.7)	40 (72.7)	38 (70.4)	43 (81.1)
**Item 3**	136 (84.0)	44 (80.0)	46 (85.2)	46 (86.8)
**Item 4**	116 (71.6)	34 (61.8)	43 (79.6)	39 (73.6)
**Item 5**	123 (75.9)	37 (67.3)	42 (77.8)	44 (83.0)
**Item 6**	139 (85.8)	46 (83.6)	46 (85.2)	47 (88.7)
**Item 7**	126 (77.8)	42 (76.4)	42 (77.8)	42 (79.2)
**Item 8**	131 (80.9)	47 (85.5)	44 (81.5)	40 (75.5)
**Item 9**	113 (69.8)	27 (49.1)	41 (75.9)	45 (84.9)
**Item 10**	134 (82.7)	41 (74.5)	46 (85.2)	47 (88.7)
**Item 11**	123 (75.9)	39 (70.9)	40 (74.1)	44 (83.0)
**Item 12**	128 (79.0)	43 (78.2)	41 (75.9)	44 (83.0)

Values are presented as *n* (%). B-SIT, Brief Smell Identification Test. Individual items are identified by item number because the proprietary test content is not reproduced.

## Data Availability

The data presented in this study are available on request from the corresponding author. The data are not publicly available due to privacy and ethical reasons.

## Supplementary Materials

The following supporting information can be downloaded at: https://www.mdpi.com/article/10.3390/children13091239/s1. Supplementary Table S1: Age-specific distribution of B-SIT total scores in children aged 6–14 years.
