# Supplementary material for: Age-Related Variation in Odor Identification Performance in Children Assessed with the Brief Smell Identification Test
Source: Children (Basel). 2026 Sep 13;13(9):1239. doi: 10.3390/children13091239 (PMC13605777; doi:10.3390/children13091239)
Supplement: Supplementary file 1 [file children-13-01239-s001.zip › children-4538370-supplementary.pdf]

Supplementary Table S1. Age-specific distribution of B-SIT total scores in children aged 6–14 years.

Age (years)	n	Mean ± SD	Median (IQR)
6	19	8.11 ± 1.33	8 (2)
7	16	8.94 ± 1.95	9 (4)
8	20	9.20 ± 1.20	9 (2)
9	19	9.21 ± 2.04	10 (3)
10	17	9.41 ± 1.33	9 (2)
11	18	9.72 ± 1.71	9.5 (3.25)
12	19	9.53 ± 1.93	10 (3)
13	15	10.13 ± 1.30	10 (2)
14	19	10.11 ± 1.41	10 (2)

Values are presented as mean ± standard deviation (SD) and median (interquartile range [IQR]). B-SIT, Brief Smell Identification Test.
